# Association between random glucose and all-cause mortality: findings from the mortality follow-up of the German National Health Interview and Examination Survey 1998

**DOI:** 10.1186/s12902-018-0319-2

**Published:** 2018-12-13

**Authors:** Jens Baumert, Christin Heidemann, Rebecca Paprott, Yong Du, Christa Scheidt-Nave

**Affiliations:** Department of Epidemiology and Health Monitoring, Robert Koch Institute, General-Pape-Strasse 62-66, D-12101 Berlin, Germany

**Keywords:** Random glucose, All-cause mortality, Fasting time, Diabetes prevention, Population-based study

## Abstract

**Background:**

Random glucose is widely measured in epidemiological studies and in the clinical setting when standardized fasting protocols and oral glucose tolerance testing or HbA_1c_ measuring are not feasible. The relationship between random glucose and all-cause mortality has hardly been studied so far and was examined in the present study.

**Methods:**

We ascertained mortality status among 5955 persons aged 18–79 years and free of known diabetes when participating in the German National Health Interview and Examination Survey 1998 (mean observation time 11.7 years, 458 deaths). Cox regression was applied to analyze the association of random serum glucose with all-cause mortality taken potential confounders into account. Relative mortality risks were estimated as hazard ratios (HRs) with 95% confidence intervals (CIs) modeling random glucose as categorical or continuous variable.

**Results:**

Compared to random glucose levels of 4.3 - < 5.3 mmol/L, HRs (95% CIs) were 1.94 (0.85–4.45) for levels < 4.3 mmol/L and 1.16 (0.89–1.50), 1.20 (0.91–1.58), 1.42 (0.88–2.29), 2.02 (1.26–3.25) and 4.71 (2.20–10.10) for levels 5.3 - < 5.8, 5.8 - < 6.8, 6.8 - < 7.8, 7.8 - < 11.1 and ≥ 11.1 mmol/L, adjusted for age, sex, lifestyle, anthropometry and chronic diseases. An additional adjustment for fasting time or HbA_1c_ yielded similar estimates. Modeling continuous random glucose by restricted cubic spline functions revealed comparable findings.

**Conclusions:**

In the present epidemiological study drawn from the general population, random glucose showed a significant association with all-cause mortality, independent of main potential confounders. Thus, random glucose measures are highly relevant to health risk assessment among people without known diabetes when fasting glucose or HbA_1c_ are difficult to obtain.

**Electronic supplementary material:**

The online version of this article (10.1186/s12902-018-0319-2) contains supplementary material, which is available to authorized users.

## Background

Diabetes and preceding states of hyperglycemia are major risk factors for cardiovascular disease (CVD) and mortality and highly prevalent among adults in Germany and worldwide [1–4]. However, findings on mortality risk vary according to the glycemic measure applied, as shown in a recent systematic review including a meta-analysis of 25 prospective studies among persons with prediabetes [5]. Numerous studies found strong increases in CVD mortality risk for elevated fasting glucose levels [2, 6, 7]. Studies investigating the association between HbA_1c_ and mortality risk among people without diagnosed diabetes have reported conflicting results, with most studies showing a J shaped association with increased risk at very low HbA_1c_ measures as well as at measures at 6.4% or above [8, 9].

Fasting glucose and HbA_1c_ are routine measures for glycemic status used for diagnosing diabetes; however, there is evidence that applying currently recommended diagnostic criteria for the diagnosis of previously unknown diabetes or prediabetes using fasting glucose and HbA_1c_ are not identifying the same people [10]. Furthermore, fasting glucose is rather difficult to obtain in large epidemiological studies and sometimes even in daily routine care, because individuals are often not in a fasting state at blood withdrawal. Moreover, different definitions for the fasting state are in use, especially in terms of fasting time, which may contribute to inconsistent glucose measurements and findings across studies [11]. HbA_1c_ assessment is connected with higher costs compared to glucose measurement.

Against this background, we examined whether random glucose as a less well-standardized but highly feasible measure is predictive of mortality from all causes among people without previously diagnosed diabetes. While it is well-known that random glucose is a strong predictor of incident diabetes [12, 13], the relationship to mortality has hardly been studied so far and did not include all-cause mortality as an outcome [14, 15]. Therefore, we asked: (1) Is there a significant relationship independent of potential confounders? and (2) Do fasting time and HbA_1c_ contribute to explain the relationship between random glucose and mortality?

## Methods

### Study design and setting

The present analysis is based on the mortality follow-up of the German National Health Interview and Examination Survey 1998 (GNHIES98) sample. The GNHIES98 was conducted by the Robert Koch Institute between October 1997 and March 1999 and included 7124 adults representative of the 18 to 79-year-old residential, non-institutionalized population in Germany. Details of the study design, sampling procedure, response rates and data collection have been published previously [16]. In brief, a two-stage cluster sampling procedure was applied for the selection of survey participants (response rate: 61.4%). In the first stage of sampling, sample points (i.e. study locations) reflecting community sizes and structures in Germany were drawn and in the second stage, random samples stratified by sex and age were selected from local population registries proportional to the sex and age structure of the population in Germany.

The survey included a standardized computer-assisted personal interview (CAPI) administered by specifically trained study physicians, a standardized self-administered questionnaire which was checked by trained interviewers for plausibility and completeness, and a physical examination, including standardized anthropometric measurements and blood sampling as well as a detailed medication review conducted by trained health professionals. Blood samples were drawn over the day and processed within one hour and stored at − 40 °C until analysis in the central laboratory unit at the Robert Koch Institute. Any medications taken in the past 7 days were recorded using the unique medication identifiers (“Pharmazentralnummer”, PZN) on the original medication containers brought to the study center by study participants for this purpose. The PZN was used for medication coding according to the WHO “Anatomical-Therapeutic-Chemical” classification system (ATC-Code).

The vital status could be assessed for 6979 among the 7124 GNHIES98 participants (98.0%) by the mortality follow-up as previously described in detail [17]. Briefly, all GNHIES98 participants who had agreed to follow-up contacts were recontacted between October 2008 and October 2011 and invited to participate in the first wave of the German Health Interview and Examination Survey for Adults (DEGS1). For participants who did not respond to the invitation, vital status was obtained from local population registries including the date of death for deceased individuals. Surviving GNHIES98 participants were censored at the last date of contact, i. e. the date of contact to the population registry for non-respondents, the date of refusal for those who actively declined participation in DEGS1, or the date of DEGS1 participation for those who also took part in DEGS1.

The GNHIES98 and its mortality follow-up were approved by the Federal Office for the Protection of Data (Germany). All participants provided written informed consent before enrolment.

### Study population

Among 7124 participants of the GNHIES98 study, a number of 6750 individuals reporting no history of physician-diagnosed diabetes and no use of antidiabetic medication within the last seven days preceding the interview were defined as free of known diabetes at the baseline examination and comprised the source population of the present study. After sequential exclusion of participants with no information regarding vital status (*n* = 138), known diabetes (*n* = 25), random glucose level or fasting time (*n* = 336) or main covariates used in the present analyses (*n* = 299), the final study population consisted of 5955 participants (2919 men and 3036 women) aged 18–79 years at baseline.

### Assessment of random glucose and fasting time

Random glucose was determined by standardized measures of serum glucose drawn from fresh whole blood specimens randomly taken over the day using glukose-oxidase-peroxidase-4-aminophenazon-phenol by a MEGA analyzer (Merck, Darmstadt, Germany). For analyses as categorical variable, random glucose level was classified in seven categories (< 4.3, 4.3 - < 5.3, 5.3 - < 5.8, 5.8 - < 6.8, 6.8 - < 7.8, 7.8 - < 11.1 and ≥ 11.1 mmol/L) as previously suggested [14]. The two highest categories followed classifications concerning oral glucose tolerance 2-h postload thresholds for assessing diabetes and impaired glucose tolerance [4].

Fasting time was defined as the difference between time of drawing blood specimen and time since the last meal was taken, the latter based on self-report. For specific analyses, fasting time was classified into the categories < 2, 2 - <  4, 4 - < 8, 8 - < 12 and ≥ 12 h.

### Assessment of covariates

Covariates were chosen a priori to control for potential confounding of the relationship between random glucose and all-cause mortality. Age, sex, educational level, smoking status, alcohol intake and physical activity were obtained by self-administered questionnaire, anthropometric measures by physical examination, history of chronic diseases by CAPI and HbA_1c_ by blood sampling.

Educational level was assessed by the Comparative Analysis of Social Mobility in Industrial Nations (CASMIN) instrument, encompassing general as well as vocational training, and classified into the categories low, medium or high [18].

Smoking status was categorized as never, former and current smoking. Alcohol intake (g/day) was obtained by a semi-quantitative food frequency questionnaire [19] and classified into no, moderate (> 0 - < 20 g/day in men, > 0 - < 10 g/day in women) and high (≥ 20 g/day in men, ≥ 10 g/day in women) alcohol intake. Physical activity was assessed by five categories as no sport, < 1 h/week, regularly 1–2 h/week, regularly 2–4 h/week, or regularly > 4 h/week. This information was aggregated into two categories (< 2 h/week or ≥ 2 h/week) for the present analysis. Body mass index (BMI) was calculated as the ratio of body weight (kg) and height squared (m^2^). History of myocardial infarction, stroke, cancer, hypertension and hyperlipidemia were chosen as chronic diseases and were each defined as no or yes.

HbA_1c_ was measured in fresh whole blood specimens with a Diamat high-performance liquid chromatography analyzer (Bio-Rad Laboratories, Munich, Germany) and reagents of Recipe (Recipe Chemicals and Instruments, Munich, Germany) in the Robert Koch Institute Central Epidemiological Laboratory [20]. For specific analyses, HbA_1c_ level was classified into < 5.0, 5.0 - < 5.7, 5.7 - < 6.5 and ≥ 6.5% as previous studies showed a U form relation of HbA_1c_ and all-cause mortality [8, 21].

### Statistical analyses

Unadjusted analyses were performed by the Rao-Scott χ^2^ test for associations between random glucose categories and categorical variables and by the *F* test for mean differences for continuous and (approximately) normally distributed variables across random glucose categories. Spearman correlation was applied to assess a potential monotonic relationship between continuous random glucose and fasting time. To display distribution measures of continuous random glucose across five fasting time categories, a Box plot was created. Crude mortality rates were calculated by dividing the number of deaths by the number of person-years observed within each random glucose category.

Adjusted analyses were performed by Cox proportional hazards regression models to estimate random glucose-specific hazard ratios (HRs) including 95% confidence intervals (95% CIs) for all-cause mortality during follow-up. As basic model, model 1 was adjusted for age (continuous) and sex. Further adjustments were made for the sociodemographic factor educational level in model 2, additionally for the lifestyle factors smoking, alcohol consumption and physical activity as well as the anthropometric factors BMI (continuous) and waist circumference (continuous) in model 3, and additionally for the five chronic diseases history of myocardial infarction, stroke, cancer, hypertension and hyperlipidemia in model 4.

First, random glucose was included in the Cox regression models as categorical variable using a level of 4.3 - < 5.3 mmol/L as reference. Secondly, random glucose was included as continuous variable in the Cox regression and modelled by a spline regression approach applying restricted cubic spline functions with four knots set at the 5th, 25th, 75th and 95th percentile and choosing the median random glucose level of 5.2 mmol/L as reference.

Sensitivity analyses were performed to assess the stability of the main analyses and comprised a) an additional adjustment for fasting time (< 2, 2 - < 4, 4 - < 8, 8 - < 12, ≥ 12 h), b) an exclusion of participants with fasting time < 2 h, c) an additional adjustment for HbA_1c_ (< 5.0, 5.0 - < 5.7, 5.7 - < 6.5 and ≥ 6.5%), d) an exclusion of participants with HbA_1c_ ≥ 6.5%, e) an exclusion of participants with follow-up time ≤ 2 years and, finally, f) an analysis in the source population (*n* = 6750) with multiple imputation of missing values using the “Fully Conditional Specification” method [22]. Furthermore, interaction analyses were carried out to examine potential modifications of the random glucose-mortality association by age, BMI and waist circumference (all continuous) and sex with adding the respective interaction terms (random glucose x modifier) to the Cox regression model 4 (described above).

Statistical analyses were performed by the statistical software package SAS version 9.4 (SAS Institute, Cary, NC, USA) using the survey procedures SURVEYMEANS, SURVEYFREQ and SURVEYPHREG to account for the complex survey design, except for analyses regarding Spearman correlation and spline modelling in the Cox regression (see below). The analyses included a survey weight accounting for deviations of the study population from the population in Germany as of December 31, 1997 within strata of sex, age, education, nationality, community size, federal state, and east/west Germany which may compensate under- or over-represented groups within these strata. Spearman correlation analyses were unweighted using CORR and spline modelling in the Cox regression was performed by PHREG which permitted consideration of weights but not accounting for cluster sampling. *P* values < 0.05 were considered to indicate statistical significance. The study followed the STROBE guidelines for cohort studies [23].

## Results

### Description of study population

Random glucose had a median level of 5.2 mmol/L and ranged from 2.6 to 20.6 mmol/L. Table 1 shows the distribution of baseline characteristics across seven random glucose categories. Participants with higher random glucose categories were significantly more often older, male, low educated, obese, current smokers, high alcohol consumers, physically inactive and reported more often a history of myocardial infarction, stroke, hypertension and hyperlipidemia compared to participants with lower random glucose categories.

**Table 1:** Baseline characteristics of the study population by random glucose categories

	Random glucose category (in mmol/L)							
Characteristic	< 4.3	4.3 - < 5.3	5.3 - < 5.8	5.8 - < 6.8	6.8 - < 7.8	7.8 - < 11.1	≥ 11.1	p value
N	224	3,105	1,510	843	161	93	19	-
Age class (%)								< 0.001
18-39 years	74.6	52.2	34.9	23.7	26.3	14.4	11.8	
40-49 years	15.2	19.9	18.5	18.7	10.6	14.0	21.8	
50-59 years	6.5	14.4	20.1	19.9	27.9	23.3	25.1	
60-69 years	2.2	9.1	15.4	24.1	21.1	28.3	23.8	
70-79 years	1.5	4.5	11.0	13.6	14.1	20.0	17.5	
Age (years)	33.4 (1.1)	41.1 (0.4)	48.0 (0.6)	51.9 (0.7)	52.1 (1.6)	57.4 (1.8)	56.5 (2.7)	< 0.001
Male sex (%)	42.6	45.3	54.0	59.3	60.6	60.2	56.9	< 0.001
Educational level (%)								< 0.001
Low	42.8	43.3	50.8	58.2	55.6	62.8	72.7	
Medium	42.8	44.3	36.6	30.1	35.0	30.9	16.3	
High	14.5	12.4	12.6	11.7	9.4	6.3	11.0	
BMI category (%)^2^								< 0.001^a^
< 25 kg/m	64.1	47.5	34.2	26.2	17.7	19.3	n.a.^a^	
25- < 30 kg/m^2^	28.9	36.2	43.5	45.7	50.4	29.1	35.7	
≥ 30 kg/m^2^	6.9	16.2	22.3	28.1	31.9	51.5	64.3	
BMI (kg/m^2^)	24.1 (0.3)	25.9 (0.1)	27.1 (0.1)	27.9 (0.2)	28.7 (0.4)	30.5 (0.7)	32.6 (1.4)	< 0.001
Waist circumference category (%)^b^								< 0.001
Normal	65.6	50.5	35.5	27.9	22.3	17.0	2.5	
Moderate	22.6	24.1	27.1	26.7	27.5	21.3	10.0	
High	11.9	25.4	37.4	45.4	50.2	61.6	87.5	
Waist circumference (cm)^c^								
Men	88.9 (1.1)	94.4 (0.4)	96.7 (0.4)	99.1 (0.6)	100.1 (1.8)	102.0 (1.5)	109.8 (3.2)	< 0.001
Women	78.4 (1.1)	81.8 (0.4)	86.8 (0.6)	90.0 (0.8)	91.5 (1.8)	99.4 (2.4)	105.9 (3.4)	< 0.001
Smoking (%)								< 0.001
Never smoker	48.3	36.9	30.4	28.7	27.4	24.9	30.3	
Former smoker	12.9	19.4	23.4	25.7	27.5	23.9	20.0	
Current smoker	38.8	43.7	46.2	45.7	45.1	51.2	49.7	
Alcohol consumption (%)^d^								< 0.001
No	21.7	18.4	18.2	18.2	16.5	27.9	9.0	
Moderate	69.8	63.9	59.2	59.7	57.7	49.9	52.4	
High	8.5	17.7	22.6	22.1	25.7	22.2	38.6	
Physical activity < 2 h/week (%)	72.8	78.9	80.9	83.3	84.6	88.2	96.5	0.003
History of chronic diseases								
Myocardial infarction	0.6	1.2	1.6	3.6	5.2	3.6	3.7	< 0.001
Stroke	0.4	0.6	1.2	1.0	0.6	6.4	4.0	< 0.001
Cancer	2.5	2.8	3.2	3.6	3.5	5.7	4.0	0.682
Hypertension	4.9	14.6	24.2	32.6	35.6	54.0	48.7	< 0.001
Hyperlipidemia	8.5	17.9	25.7	28.8	33.2	38.6	55.5	< 0.001

Information is given as arithmetic mean (standard error) or percentage. Differences in means or percentages by random glucose categories were assessed by F test or Rao-Scott χ^2^ test

^a^ no observations in this cell; p value for association excluding random glucose level ≥ 11.1 mmol/L

^b^ normal / moderate / high: < 94 / 94 - < 102 / ≥ 102 cm in men and < 80 / 80 - < 88 / ≥ 88 cm in women

^c^ mean (standard error) is given separately for men and women due to sex-specific differences in waist circumference distribution

^d^ no / moderate / high: 0 / > 0 - < 20 / ≥ 20 g/day in men and 0 / > 0 - < 10 / ≥ 10 g/day in women

### Association of random glucose and fasting time

**Fig. 1:**
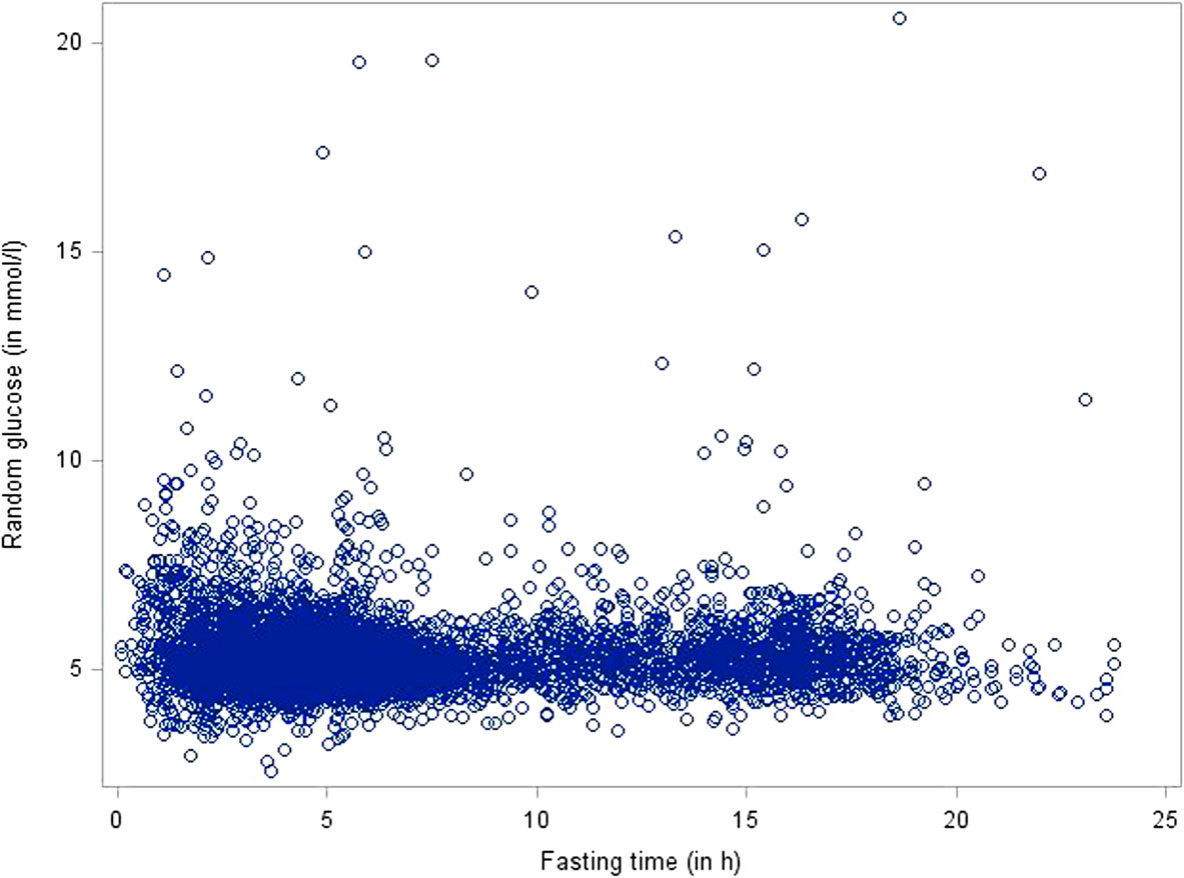
Correlation of random glucose and fasting time, displayed as scatter plot

Median fasting time was 5.4 h with lower quartile 3.8 h and upper quartile 8.6 h. Continuous random glucose level and fasting time were weakly and non-monotonically correlated (Spearman’s *ρ* = − 0.044) as shown by a scatter plot (Fig. 1).

A Box plot revealed comparable distribution measures for random glucose levels across five fasting time categories (Additional file 1: Figure S1); slightly higher =median random glucose (5.4 mmol/L) and higher variation in glucose measures was observed among persons with lowest fasting time (< 2 h) as compared to those with fasting times between 2- < 4 h and ≥ 12 h.

### Association of random glucose and all-cause mortality

A total number of 458 deaths (men: 287, women: 171) was observed over a mean follow-up time of 11.7 years (standard error 0.1). Crude mortality rates per 1000 person-years ranged from 3.8 in the lowest to 41.2 in the highest random glucose category (Table 2).

**Table 2:** Mortality rate and risk for all-cause mortality (HR (95% CI)) by random glucose categories

	Random glucose category (in mmol/L)						
	< 4.3	4.3 - < 5.3	5.3 - < 5.8	5.8 - < 6.8	6.8 - < 7.8	7.8 - < 11.1	≥ 11.1
N	224	3105	1510	843	161	93	19
N of deaths	10	158	124	110	27	22	7
Crude MR^a^	3.8	4.0	8.1	11.8	13.4	25.8	41.2
Model 1	2.46 (1.12–5.38)	ref.	1.06 (0.84–1.34)	1.18 (0.93–1.51)	1.34 (0.83–2.15)	1.85 (1.15–2.98)	4.36 (2.00–9.50)
Model 2	2.43 (1.11–5.34)	ref.	1.06 (0.84–1.35)	1.18 (0.93–1.51)	1.29 (0.80–2.08)	1.77 (1.08–2.90)	4.45 (2.07–9.55)
Model 3	2.11 (1.00–4.43)	ref.	1.14 (0.90–1.46)	1.22 (0.94–1.59)	1.43 (0.90–2.26)	1.79 (1.07–3.01)	4.42 (2.14–9.11)
Model 4	1.94 (0.85–4.45)	ref.	1.16 (0.89–1.50)	1.20 (0.91–1.58)	1.42 (0.88–2.29)	2.02 (1.26–3.25)	4.71 (2.20–10.10)

^a^ *MR: crude mortality rate per 1000 person-years*

*model 1: adjusted for age and sex*

*model 2: adjusted for age, sex and educational level*

*model 3: adjusted for age, sex, educational level, body mass index, waist circumference, smoking, alcohol consumption and physical* *activity*

*model 4: adjusted for age, sex, educational level, body mass index, waist circumference, smoking, alcohol consumption, physical*

*activity, myocardial infarction, stroke, cancer, hypertension and hyperlipidemia*

Compared to participants with random glucose levels in the reference category (4.3 - < 5.3 mmol/L), the age- and sex-adjusted relative mortality risk estimated by Cox regression was significantly increased for participants in the lowest random glucose category (< 4.3 mmol/L) as well as for those with random glucose levels in the two highest categories (model 1, Table 1) which persisted after further adjustment for educational level, lifestyle and anthropometry (model 3). Additional adjustment for major chronic diseases attenuated the relative mortality risk (HR: 1.94, 95% CI: 0.85–4.45) for participants in the lowest random glucose category to non-significance, but not for those in the higher categories with HRs (95% CI) of 1.16 (0.89–1.50), 1.20 (0.91–1.58), 1.42 (0.88–2.29), 2.02 (1.26–3.25) and 4.71 (2.20–10.10) for levels of 5.3 - < 5.8 to ≥11.1 mmol/L (model 4).

**Fig. 2:**
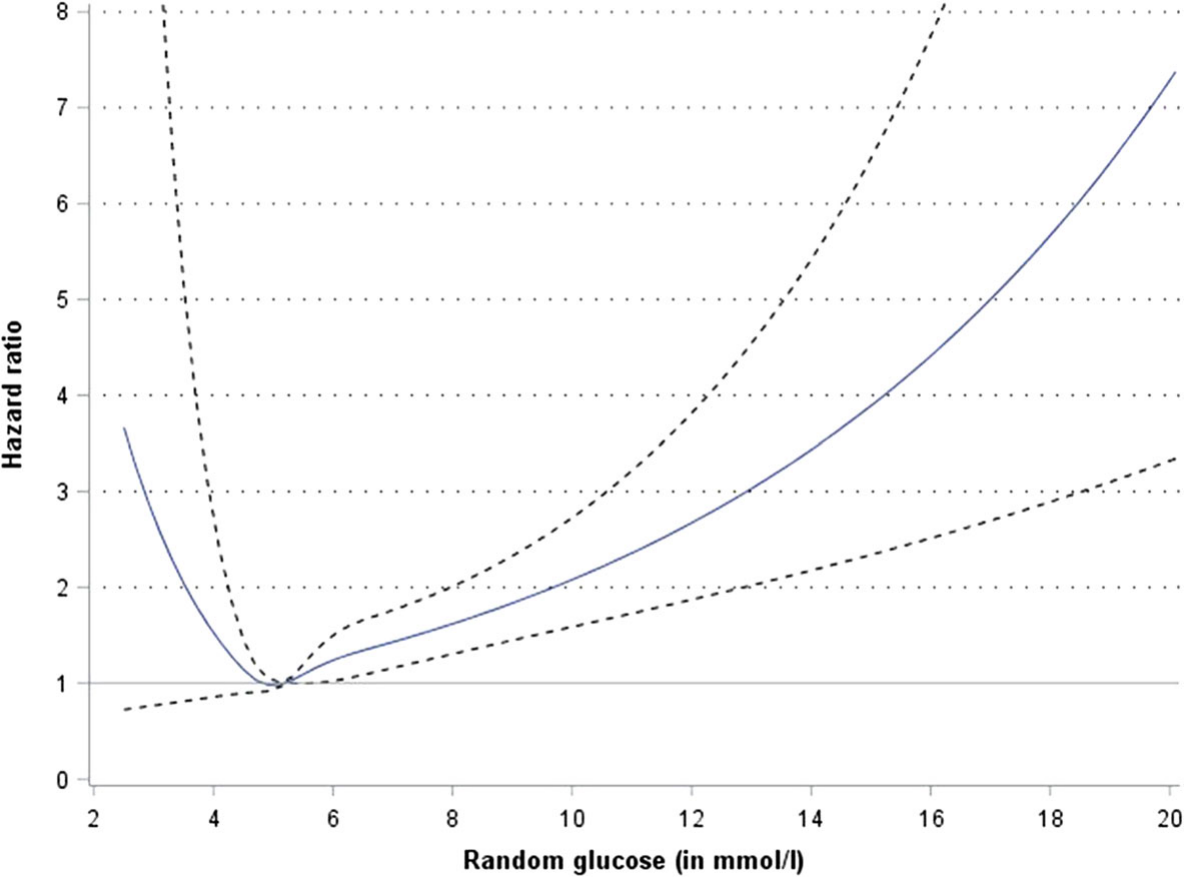
Risk for all-cause mortality (95% CI) by continuous random glucose level, estimated by Cox regression and applying restricted cubic spline functions for modelling random glucose level

Modeling continuous random glucose levels by restricted cubic spline functions in the Cox regression models revealed a similar shape of the random glucose-mortality association as shown for model 4 (Fig. 2). However, the confidence intervals at the tails of the hazard ratios were wide due to the low number of participants with extreme random glucose values.

### Sensitivity analyses

Additional adjustment for fasting time in model 4 or excluding participants with fasting time < 2 h did not change estimates substantially (Table 3a and b). Hazard ratios for the two highest categories were attenuated but remained significantly increased when additionally controlling for HbA_1c_ (Table 3c). After excluding participants with HbA_1c_ ≥ 6.5%, no more observations in the highest random glucose category were observed; however, the relative risk of death was significantly increased among persons with random glucose levels of 7.8 - < 11.1 mmol/L (Table 3d). Comparable estimates as in the main analyses were obtained when participants with follow-up time ≤ 2 years were excluded (Table 3e). Performing the main analyses in the source population (*n* = 6750) with imputation of missing variable information estimated significant hazard ratios for the three highest categories; the hazard ratio for random glucose level ≥ 11.1 mmol/L was lower than in the analyses using the study population (2.96 versus 4.71) (Table 3f).

**Table 3:** Sensitivity analyses for risk for all-cause mortality (HR (95% CI)) by random glucose categories

	Random glucose category (in mmol/L)						
Model	< 4.3	4.3 - < 5.3	5.3 - < 5.8	5.8 - < 6.8	6.8 - < 7.8	7.8 - < 11.1	≥ 11.1
a) adjusted for fasting time^a^	1.96 (0.87–4.43)	ref.	1.16 (0.89–1.51)	1.22 (0.92–1.60)	1.46 (0.89–2.39)	2.10 (1.28–3.42)	4.99 (2.33–10.73)
b) excluding fasting time < 2 h^b^	2.07 (0.85–5.03)	ref.	1.14 (0.87–1.50)	1.18 (0.89–1.57)	1.43 (0.85–2.41)	2.20 (1.25–3.86)	4.38 (2.01–9.58)
c) adjusted for HbA_1c_^c^	1.96 (0.86–4.44)	ref.	1.17 (0.90–1.51)	1.21 (0.91–1.60)	1.35 (0.83–2.20)	1.82 (1.09–3.03)	3.46 (1.49–8.04)
d) excluding HbA_1c_ ≥ 6.5%^d^	1.73 (0.73–4.09)	ref.	1.12 (0.85–1.48)	1.23 (0.92–1.63)	1.08 (0.54–2.13)	2.46 (1.50–4.03)	–
e) excluding FU time ≤ 2 years^e^	2.10 (0.87–5.06)	ref.	1.12 (0.85–1.46)	1.11 (0.82–1.48)	1.30 (0.77–2.19)	1.97 (1.21–3.21)	3.72 (1.51–9.19)
f) multiple imputation^f^	1.68 (0.92–3.07)	ref.	1.12 (0.88–1.41)	1.25 (0.98–1.59)	1.81 (1.17–2.80)	1.94 (1.26–3.01)	2.96 (1.48–5.92)

*Models with adjustment as in model 4 with additional adjustment for fasting time in a) and HbA*_*1c*_ *in c)*

^a^ *fasting time with five categories (< 2, 2 - < 4, 4 - < 8, 8 - < 12 and ≥ 12 h)*

^b^ *n = 5579 (428 cases, 5151 non-cases)*

^c^ *HbA*_*1c*_ *with five categories (< 5.0, 5.0- < 5.7, 5.7- < 6.5, ≥ 6.5%)*

^d^ *n* = *5718 (386 cases, 5332 non-cases), no participant with random glucose ≥ 11.1 mmol/L*

^e^ *n = 5912 (415 cases, 5497 non-cases)*

f *n = 6570 (551 cases, 6061 non-cases, 138 missings), missings were replaced in the source population using multiple imputation by fully conditional specification*

### Interaction analyses

No significant modifications in the association of random glucose level category and all-cause mortality was observed by age, sex and BMI in interaction analyses (p for interactions 0.208, 0.744 and 0.108). However, a significant interaction was estimated regarding waist circumference (p for interaction 0.011); the association was more pronounced in participants with higher than with lower waist circumference levels. Modelling random glucose level by restricted cubic spline functions revealed no significant interaction by age, sex, BMI or waist circumference (p for interactions 0.133, 0.156, 0.802 and 0.410).

## Discussion

### Overall findings

In the present epidemiological study drawn from the general population, random glucose was significantly associated with all-cause mortality, as an increase in mortality risk was found in participants with very low and high random glucose levels which was independent from main mortality-related risk factors as well as from fasting time and HbA_1c_. Changes in the effects of random glucose levels on mortality risk were rather low when adjusting for these factors. As far as we know, this is the first investigation about the association of random glucose and all-cause mortality in a study population drawn from the general population.

### Association of random glucose and all-cause mortality

The present study investigated the association of random glucose with all-cause mortality, in contrast to a variety of previous studies which examined other endpoints. A large study based on a population-based sample of Chinese adults found a monotonically increasing CVD mortality risk using the same random glucose categories [14]; in contrast to our study with endpoint all-cause mortality, CVD mortality risks here were almost equal in the two lowest categories. Rosness et al. studied the association of random glucose (categorical and continuous) with dementia-related mortality in two study populations from Norway with different age ranges (35–49 and 65–80 years) and observed a relation in the younger but not in the older age group [15]; random glucose was categorized in quintiles as well as modelled with spline functions.

Both studies above did not show a U or J shaped association which may be due to different mortality endpoints (CVD or dementia-related in contrast to all-causes). However, in a large collaborative investigation including 48 studies with about 33,000 cases of death, a J shaped association of fasting glucose and death from all causes was reported in subjects without known diabetes and known pre-existing cardiovascular disease at baseline [2]. In a previous study regarding HbA_1c_ as another glycemic marker using the same data source (GNHIES98) as in the present analyses, Paprott et al. revealed a U shaped HbA_1c_-mortality relation when HbA_1c_ was modelled with spline functions in subjects without known diabetes indicating that very low HbA_1c_ levels are associated with increased risk for all-cause mortality, additionally to high HbA_1c_ levels (≥ 6.4%) [8]. This is in line with findings of a meta-analysis of observational studies which indicated a J shaped relationship between HbA_1c_ levels and all-cause mortality in the non-diabetic as well as in the diabetic population [9].

The association of random glucose and all-cause mortality was independent of main other mortality-related risk factors. The changes in the hazard ratios when sociodemographic and lifestyle factors as well as chronic diseases were taken into account was rather low in the two highest random glucose categories indicating no substantial confounding in the effects on all-cause mortality by these factors. In contrast, the effect change for the lowest random glucose category (< 4.3 mmol/L) by these adjustments was more pronounced especially when chronic diseases were included in the regression model. Thus, the increased mortality risk in the very low compared to the reference random glucose group may partly be explained by these unfavourable conditions as previously found for low HbA_1C_ levels [8]. However, as the number of events in the lowest random glucose category was only 10, this finding should not be overinterpreted; larger studies are necessary to examine the relation of very low random glucose levels and all-cause mortality in more detail.

Our interaction analyses suggest that an increased mortality risk in low and high random glucose levels was more pronounced in participants with higher compared to lower waist circumference levels. Previously, an increased risk for all-cause mortality has been shown for elevated levels of waist circumference in a population-based study of Canadian adults [24]. Considering this finding in the face with our interaction analysis may indicate that especially a combination of unfavourable levels in random glucose (low and high) and waist circumference (elevated) is related to an increased mortality risk which was surprising and may deserve further attention in future and larger studies.

However, the low case numbers at both tails of the random glucose level distribution should be kept in mind here and therefore interpretation regarding the interaction findings in the present study should be taken with caution.

### Random glucose and fasting time

In the present study, the correlation between random glucose and fasting time was weak (correlation coefficient − 0.04); the distribution of random glucose was rather comparable across five fasting time categories, however a non-monotonic and suggestive J shaped association could be observed as shown in Additional file 1: Figure S1. This finding may be initially surprising as considerable more pronounced differences would be expected. However, Moebus et al. reported mean random glucose values of 5.3 mmol/L at zero hours, 5.2 mmol/l at two hours and 5.0 mm/L at 8 h since last caloric intake in a study of about 28,000 primary care patients [11] and that higher mean random glucose levels are mainly seen in the first 3 h after a caloric intake which is comparable to our findings. A study of non–glucose-intolerant adults (i.e. also without diabetes) with existing CVD drawn from the Framingham Heart Study (FHS) showed only little differences in age- and sex-adjusted percentiles between random glucose and fasting glucose [25].

In our study, additional adjustment for fasting time did not alter substantially the random glucose-mortality relation. Regarding other endpoints, a study from Asia and the Pacific region had data on both random and fasting glucose and revealed weaker estimates regarding stroke but comparable estimates regarding ischemic heart disease (IHD) for random compared to fasting glucose [26].

### Limitations and strengths

Our study has some limitations which are described in the following. First, although the study population and the overall number of fatal cases were large, the number of cases was rather low for very low and high glucose levels leading to wide confidence intervals; thus, findings should be interpreted with caution for random glucose values at the tails of the distribution. Second, random glucose was measured in serum in contrast to several other studies where blood plasma was used. However, glucose values measured in serum or plasma seem to differ only slightly with lower values in serum which may be considered as not physiologically relevant [27]. Third, random glucose was measured only at the baseline examination; thus potential changes in random glucose levels at the individual level per participant during the follow-up time could not be assessed. Fourth, though non-response in our study was addressed by using survey weights which may compensate under-represented groups (e.g. older aged or low educated), a possible selection bias due to non-response cannot be excluded. Fifth, about 12% of participant had to be excluded due to missing information in at least one of used variables which may lead also to selection bias. However, a sensitivity analysis applying multiple imputation revealed comparable findings as in the main analyses indicating a rather low selection bias due to missing information. Sixth, residual confounding cannot be excluded, especially regarding further mortality-related risk factors and subclinical diseases which could not be taken into account in the present analyses.

A major strength of the present study is that it was based on a large population-based sample representative of the adult population in Germany with standardized and quality controlled data ascertainment. The mortality follow-up was almost complete with only 2% of participants lost to follow-up; furthermore, the mean follow-up time of almost 12 years was rather long.

## Conclusions

The present study conducted in a nationally representative study population indicated a significant association of random glucose levels with all-cause mortality in adults without known diabetes, even after controlling for mortality-related risk factors, HbA_1c_ and fasting time. Surprisingly, the correlation between random glucose level and fasting time was non-monotonic and weak.

Undoubtedly, measuring fasting glucose or HbA_1c_ have the advantage of being more precise in assessing diabetes risk and diagnosis. However, regarding the value for the assessment of mortality risks in epidemiological studies with large sample size, fasting glucose, HbA_1c_ and random glucose seem to be comparable. The advantage of random glucose consists in its easy assessment independent of the fasting state in contrast to fasting glucose and is connected with lower costs compared to HbA_1c_ which makes it a suitable marker in specific situations. This is particularly true for large population-based health studies, since fasting blood sampling after an overnight fasting period may jeopardize participation rates.

In conclusion, our findings add to existing evidence that random glucose is a useful tool for assessing health risks among people without diagnosed diabetes, especially when fasting glucose or HbA_1c_ is difficult to obtain.

## Additional file

Additional file 1: **Figure S1.** The box plot gives information about distribution measures of random glucose levels stratified by fasting time categories: the full range from minimum to maximum (at bottom and top of the whiskers), the interquartile range from lower to upper quartile (at bottom and top of the boxes), the median (straight line inside the boxes) and the mean (point inside the boxes); the median values in the fasting time categories are connected by a solid line. (DOCX 30 kb)

## Abbreviations

BMI: Body mass index
CAPI: Computer-assisted personal interview
CASMIN: Comparative Analysis of Social Mobility in Industrial Nations
CI: Confidence interval
CVD: Cardiovascular disease
DEGS1: German Health Interview and Examination Survey for Adults
FHS: Framingham Heart Study
GNHIES98: German National Health Interview and Examination Survey 1998
HbA_1c_: Glycated hemoglobin A_1c_
HR: Hazard ratio
RKI: Robert Koch Institute

## References

[1] van Dieren S, Beulens JW, van der Schouw YT, Grobbee DE, Neal B (2010). The global burden of diabetes and its complications: an emerging pandemic. Eur J Cardiovasc Prev Rehabil.

[2] Rao Kondapally Seshasai S, Kaptoge S, Thompson A, Di Angelantonio E, Gao P, Sarwar N (2011). Diabetes mellitus, fasting glucose, and risk of cause-specific death. N Engl J Med.

[3] Tancredi M, Rosengren A, Svensson AM, Kosiborod M, Pivodic A, Gudbjornsdottir S (2015). Excess mortality among persons with type 2 diabetes. N Engl J Med.

[4] World Health Organization (2016). Gobal report on diabetes.

[5] Huang Y, Cai X, Mai W, Li M, Hu Y (2016). Association between prediabetes and risk of cardiovascular disease and all cause mortality: systematic review and meta-analysis. BMJ.

[6] Sarwar N, Gao P, Seshasai SR, Gobin R, Kaptoge S, Emerging Risk Factors Collaboration (2010). Diabetes mellitus, fasting blood glucose concentration, and risk of vascular disease: a collaborative meta-analysis of 102 prospective studies. Lancet.

[7] Park C, Guallar E, Linton JA, Lee DC, Jang Y, Son DK (2013). Fasting glucose level and the risk of incident atherosclerotic cardiovascular diseases. Diabetes Care.

[8] Paprott R, Schaffrath Rosario A, Busch MA, Du Y, Thiele S, Scheidt-Nave C (2015). Association between hemoglobin A1c and all-cause mortality: results of the mortality follow-up of the German National Health Interview and examination survey 1998. Diabetes Care.

[9] Cavero-Redondo I, Peleteiro B, Alvarez-Bueno C, Rodriguez-Artalejo F, Martinez-Vizcaino V (2017). Glycated haemoglobin A1c as a risk factor of cardiovascular outcomes and all-cause mortality in diabetic and non-diabetic populations: a systematic review and meta-analysis. BMJ Open.

[10] NCDRF collaboration (2015). Effects of diabetes definition on global surveillance of diabetes prevalence and diagnosis: a pooled analysis of 96 population-based studies with 331,288 participants. Lancet Diabetes Endocrinol.

[11] Moebus S, Gores L, Losch C, Jockel KH (2011). Impact of time since last caloric intake on blood glucose levels. Eur J Epidemiol.

[12] Kowall B, Rathmann W, Giani G, Schipf S, Baumeister S, Wallaschofski H (2013). Random glucose is useful for individual prediction of type 2 diabetes: results of the study of health in Pomerania (SHIP). Prim Care Diabetes.

[13] Bowen ME, Xuan L, Lingvay I, Halm EA (2015). Random blood glucose: a robust risk factor for type 2 diabetes. J Clin Endocrinol Metab.

[14] Bragg F, Li L, Bennett D, Guo Y, Lewington S, Bian Z (2016). Association of Random Plasma Glucose Levels with the risk for cardiovascular disease among Chinese adults without known diabetes. JAMA Cardiol.

[15] Rosness TA, Engedal K, Bjertness E, Strand BH (2016). Association between random measured glucose levels in middle and old age and risk of dementia-related death. J Am Geriatr Soc.

[16] Thefeld W, Stolzenberg H, Bellach BM. [The Federal Health Survey: response, composition of participants and non-responder analysis]. Gesundheitswesen 1999;61 Spec No:S57–S61. 10726397

[17] Wolf IK, Busch M, Lange M, Kamtsiuris P, Doelle R, Richter A (2014). Mortality follow-up of the German health interview and examination survey for adults (DEGS) : methods and first results. Bundesgesundheitsblatt Gesundheitsforschung Gesundheitsschutz.

[18] Brauns HSS, Steinmann S, Hoffmeyer-Zlotnik JHWC (2003). The CASMIN educational classification in international comparative research. Advances in cross-National Comparison.

[19] Burger M, Mensink G, Bronstrup A, Thierfelder W, Pietrzik K (2004). Alcohol consumption and its relation to cardiovascular risk factors in Germany. Eur J Clin Nutr.

[20] Heidemann C, Scheidt-Nave C, Richter A, Mensink GB (2011). Dietary patterns are associated with cardiometabolic risk factors in a representative study population of German adults. Br J Nutr.

[21] Aggarwal V, Schneider AL, Selvin E (2012). Low hemoglobin a(1c) in nondiabetic adults: an elevated risk state?. Diabetes Care.

[22] Berglund P (2015). Multiple imputation using the fully conditional specification method: a comparison of SAS® Stata, IVEware, and R.

[23] von Elm E, Altman DG, Egger M, Pocock SJ, Gotzsche PC, Vandenbroucke JP (2008). The strengthening the reporting of observational studies in epidemiology (STROBE) statement: guidelines for reporting observational studies. J Clin Epidemiol.

[24] Staiano AE, Reeder BA, Elliott S, Joffres MR, Pahwa P, Kirkland SA (2012). Body mass index versus waist circumference as predictors of mortality in Canadian adults. Int J Obes.

[25] Port SC, Goodarzi MO, Boyle NG, Jennrich RI (2005). Blood glucose: a strong risk factor for mortality in nondiabetic patients with cardiovascular disease. Am Heart J.

[26] Lawes CM, Parag V, Bennett DA, Suh I, Lam TH, Whitlock G (2004). Blood glucose and risk of cardiovascular disease in the Asia Pacific region. Diabetes Care.

[27] Frank EA, Shubha MC, D'Souza CJ (2012). Blood glucose determination: plasma or serum?. J Clin Lab Anal.

