# Supplementary material for: Association between random glucose and all-cause mortality: findings from the mortality follow-up of the German National Health Interview and Examination Survey 1998
Source: BMC Endocr Disord. 2018 Dec 13;18:95. doi: 10.1186/s12902-018-0319-2 (PMC6293613; doi:10.1186/s12902-018-0319-2)
Supplement: Supplementary file 1 — Figure S1. The box plot gives information about distribution measures of random glucose levels stratified by fasting time categories: the full range from minimum to maximum (at bottom and top of the whiskers), the interquartile range from lower to upper quartile (at bottom and top of the boxes), the median (straight line inside the boxes) and the mean (point inside the boxes); the median values in the fasting time categories are connected by a solid line. (DOCX 30 kb) [file 12902_2018_319_MOESM1_ESM.docx]

**Additional file 1: Figure S1** Distribution of random glucose level stratified by fasting time category, displayed as Box plot


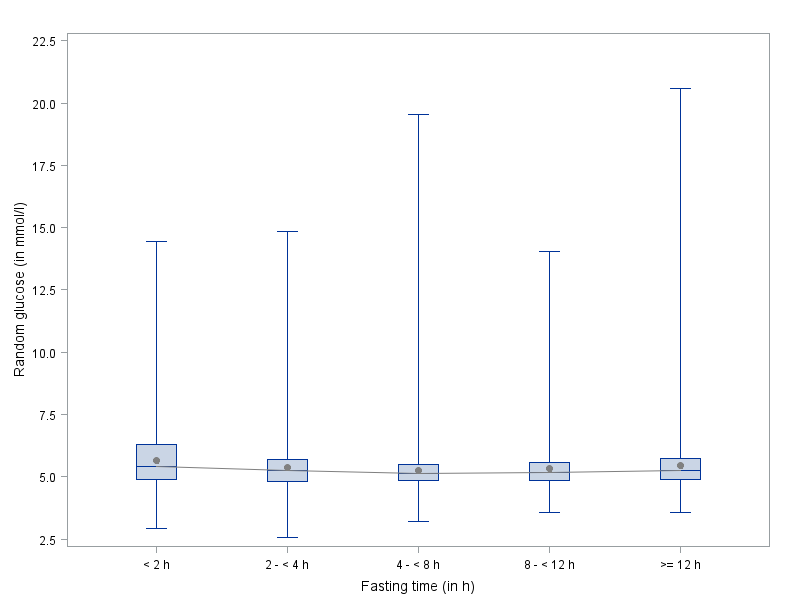


*The box plot gives information about distribution measures of random glucose levels stratified by fasting time categories: the full range from minimum to maximum (at bottom and top of the whiskers), the interquartile range from lower to upper quartile (at bottom and top of the boxes), the median (straight line inside the boxes) and the mean (point inside the boxes); the median values in the fasting time categories are connected by a solid line*
